# Quantifying the importance of inhaler attributes corresponding to items in the patient satisfaction and preference questionnaire in patients using Combivent Respimat

**DOI:** 10.1186/s12955-017-0780-z

**Published:** 2017-10-16

**Authors:** Kimberly H. Davis, Jun Su, Juan Marcos González, Jeremiah J. Trudeau, Lauren M. Nelson, Brett Hauber, Kelly A. Hollis

**Affiliations:** 1 RTI Health Solutions, 3040 Cornwallis Road, Post Office Box 12194, Research Triangle Park, Durham, NC 27709-2194 UK; 2 Boehringer Ingelheim Pharmaceuticals, Inc., 900 Ridgebury Road, AOB 1C180, Ridgefield, CT 06877 USA; 3 Bioverativ, 225 Second Avenue, Waltham, MA 02451 USA; 4 Duke Clinical Research Institute, Durham, NC 27705 UK

**Keywords:** Best-worst scaling, Chronic obstructive pulmonary disease, Inhaler attributes, Preference, Satisfaction

## Abstract

**Background:**

Physicians consider ease of use, satisfaction, and preferences when prescribing an inhaler device. These factors may impact appropriate usage and compliance.

**Methods:**

The objectives were to quantify the relative importance of inhaler attributes in patients currently using Combivent Respimat by eliciting preferences for performance and convenience attributes assessed by items in the Patient Satisfaction and Preference Questionnaire (PASAPQ).

Using a pharmacy database, 19,964 adults in the United States who filled ≥2 Combivent Respimat prescriptions were identified. Of those, 8150 patients were randomly selected to receive invitation letters. The online cross-sectional survey included the PASAPQ and best-worst scaling (BWS) questions. The PASAPQ measures satisfaction with medication attributes across two domains: performance and convenience. BWS questions asked participants to select the most and least important device attributes. A descriptive statistics analysis of the PASAPQ and a random-parameters logit model of BWS responses were conducted.

**Results:**

The survey was completed by 503 participants. Most were female (57.3%), white (88.5%), and 51–70 years old (67.6%). Approximately 47% reported a chronic obstructive pulmonary disease diagnosis, 21.9% asthma, 8.2% other lung disease, and 23.1% more than one lung disease. PASAPQ scores indicated that the majority were satisfied or very satisfied; up to 20% reported being dissatisfied with Combivent Respimat. The three most important inhaler attributes were *Feeling that your medicine gets into your lungs*, *Inhaler works reliably*, and *Inhaler makes inhaling your medicine easy.* The most important attributes corresponded to six of seven items in the PASAPQ performance domain.

**Conclusions:**

Most participants reported satisfaction with Combivent Respimat. Performance attributes were more important than convenience attributes.

## Background

Satisfaction with an inhaler device may predict treatment continuance, appropriate medication use, and compliance in patients with lower respiratory conditions [1, 2]. Among patients and health care professionals, ease of use and patients’ preference for a specific inhaler device are considered important factors when prescribing an inhaler [3–5]. In August 2012, the Combivent Respimat soft mist inhaler, a novel, propellant-free inhaler, was approved by the Food and Drug Administration for the treatment of chronic obstructive pulmonary disease (COPD) [6]. It is important to measure satisfaction and preference for inhaler device features as part of the post-marketing period for a new inhaler device in a real-world context.

A systematic literature review of 20 studies explored links between treatment satisfaction and adherence, and/or persistence with prescribed treatments among patients with multiple diseases or disorders [7]. The findings suggested that greater treatment satisfaction is associated with improved compliance and persistence with medication.

Another systematic literature review identified 29 studies that included preference and satisfaction measurement with inhaler devices [8]. Most studies used a direct preference question, questions with open-ended responses, or nonvalidated proprietary questionnaires to assess patient satisfaction and preferences. The review identified only two questionnaires that were developed and have undergone psychometric evaluation: the Patient Device Experience Assessment and the Patient Satisfaction and Preference Questionnaire (PASAPQ) [8].

The PASAPQ was developed specifically to measure satisfaction and preference with any type of inhaler device, and the questionnaire does not reference the medication that the device delivers [9]. The process of PASAPQ development included a literature review, patient focus groups, expert opinion, and field testing to assess the validity, reliability, and responsiveness and to define a minimally important difference [10, 11]. Details on the development and validation of the PASAPQ have been published elsewhere [10, 11]. The development of the PASAPQ is consistent with 2009 Food and Drug Administration Patient-Reported Outcome guidelines [12]. Recently, a few patient-reported instruments were developed to measure various aspects of inhaler attributes in patients with COPD and/or asthma [3, 13, 14]. However, none were designed to measure the importance of specific attributes associated with inhaler devices.

Some researchers suggest that satisfaction implies preference [8, 15]. However, in the disciplines of cognitive psychology and economics, preference and satisfaction can be viewed as different constructs. Measuring preferences entails asking people to compare the subjective value expected from an outcome to the subjective value expected from a different outcome. Measuring satisfaction involves asking people to assess an actual experience relative to their expectation for an outcome [16]. In other words, satisfaction measures shed light on what proportion of the maximum outcome value is experienced, while preferences shed light on the relative value people place on multiple outcomes.

Consider, for example, that a patient was asked to use a new inhaler, and the patient reported experiencing a decrease in her satisfaction with one aspect of the inhaler and an equal increase in her satisfaction with another aspect, so the patient’s overall satisfaction scores for the new and old inhalers were identical. To determine whether the use of the new inhaler will change (positively or negatively) the patients’ well-being, additional information on her preference, or relative value of each inhaler attribute, would need to be gathered. Hence, both pieces of information are important in understanding the impact of treatment-related outcomes on patients’ well-being. Not gathering preference information for satisfaction results is equivalent to assuming that all items of a satisfaction instrument are equally important (weighted) and equally impactful. This study elicits both satisfaction and relative preferences for aspects of a single inhaler type in an effort to assess the differences between these constructs when participants evaluate similar experiences with an inhaler.

In the context of this study, measuring preference was an opportunity to understand how each participant valued inhaler attributes relative to each other; while measuring satisfaction using a comparable set of items was an opportunity to assess the value each participant placed on the experience of using the Combivent Respimat. Satisfaction data specific to the Combivent Respimat inhaler have been collected using the PASAPQ in previous clinical trials [1, 9, 17]. The primary objective of this study was to quantify patients’ preferences for performance and convenience attributes assessed by items in the PASAPQ and to test for differences in these preferences among participants in a real-world observational setting.

## Methods

This cross-sectional survey was conducted among patients in the United States who had filled at least two prescriptions for Combivent Respimat between March 1, 2013, and August 31, 2013. The survey was closed once the targeted sample size (*N* = 500) was reached. Patients were identified through VisibilityRx, which maintains a large national retail pharmacy database of dispensed prescriptions [18]. Potentially eligible patients were invited to complete the survey via a direct mail invitation letter from their pharmacies. The invitations included a link to a secure website to access the survey questionnaire. Following electronic consent, eligible patients were given access to the questionnaire after meeting the study criteria.

All patients were identified and recruited for participation in the survey via VisibilityRx’s pharmacy network, following a process compliant with the Health Insurance Portability and Accountability (HIPAA) Act of 1996 [19]. All study documents, including the initial invitation letter and informed consent form, were approved by RTI International’s institutional review board.

### Survey questionnaire

The questionnaire included screener questions, questions related to demographics and clinical information, the PASAPQ, and best-worst scaling (BWS) questions. Two screener questions assessed the participant’s age and the number of Combivent Respimat prescriptions that the participant had filled at a pharmacy over the previous 8 months, which reflects the time frame that Combivent Respimat was readily available in pharmacies.

#### Patient satisfaction and preference questionnaire

The 14-item version of the PASAPQ was used in the survey to measure patient satisfaction that includes a performance domain (7 items), a convenience domain (6 items), and an overall satisfaction question (item 14) (Table 1). A total score is derived from a summary of the first 13 questions. Each PASAPQ question has Likert-type response options of 1 (very dissatisfied) to 7 (very satisfied). The responses on the total score of the PASAPQ and the two PASAPQ domain scores are scored using the algorithm developed by Monz and colleagues and transformed on 0-to-100–point scales, with higher scores indicating greater satisfaction [10].

**Table 1:** Patient satisfaction and preference questionnaire

Domain	Question	Description
Performance	Q1	Overall feeling of inhaling
	Q2	Inhaled dose goes to lungs
	Q3	Amount of medication left
	Q4	Works reliably
	Q5	Ease of inhaling a dose
	Q10	Using the inhaler
	Q11	Speed medicine comes out
Convenience	Q6	Instructions for use
	Q7	Size of inhaler
	Q8	Durability of inhaler
	Q9	Ease of cleaning inhaler
	Q12	Ease of holding during use
	Q13	Convenience of carrying
Stand Alone	Q14	Overall satisfaction

The total score includes performance and convenience domain scores

#### Best-worst scaling questions

**Fig. 1:**
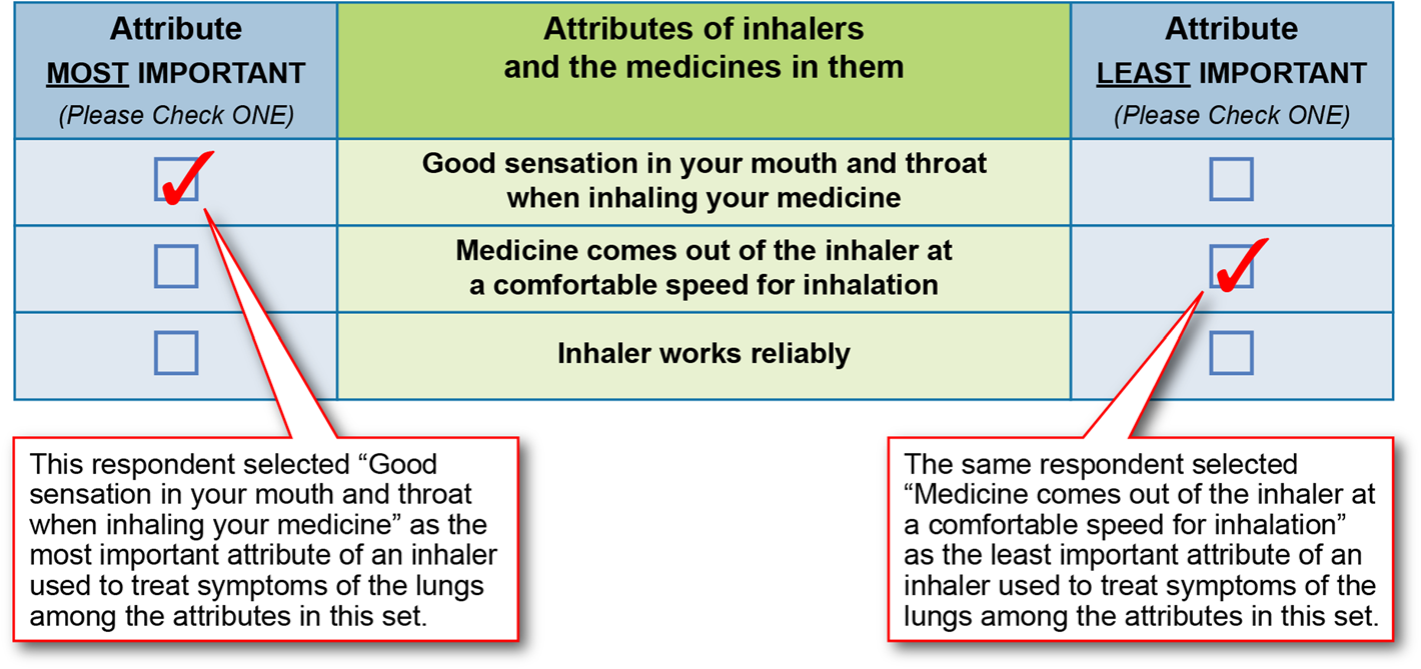
Example of a Best-Worst Scaling Question

Preferences were elicited using object-case BWS. This type of preference elicitation is designed to quantify relative importance weights for a set of attributes or outcomes. Participants are asked to evaluate a series of subsets of the total set of attributes and to indicate which item in the subset is best (or most important) and which attribute in the subset is worst (or least important) [20–22]. The pattern of responses over the series of BWS questions yields the relative desirability or importance of each attribute [20–22]. The inhaler attributes in this study were derived from the items assessed in the PASAPQ. The full set of attributes is composed of 12 of the 13 items in the performance and convenience domains of the PASAPQ. Inhaler size, an item in the PASAPQ performance domain, was deleted from the study based on feedback received from patients during the pretest interviews (see section below). PASAPQ item 14, which measures overall satisfaction with an inhaler, was not included in the list of attribute definitions because it does not characterize a specific inhaler attribute. Table 2 presents the final set of attributes included in the BWS questions and the attribute definitions provided to participants. Figure 1 displays an example of a BWS question.

**Table 2:** Final set of attributes used in the best-worst scaling questions

Attribute	Definitions
Good sensation in your mouth and throat when inhaling your medicine	With this attribute, we would like you to think about how important it is to have an inhaler with a medicine that gives you a good overall sensation in your mouth and throat when inhaling the medicine.
Feeling that your medicine gets into your lungs	With this attribute, we would like you to think about how important it is to have an inhaler with a medicine that you feel gets fully into your lungs after inhaling it.
Being able to tell how much medicine is left	With this attribute, we would like you to think about how important it is to have an inhaler that tells you how much medicine is left in the inhaler and has an indicator that lets you know when you need to refill your prescription.
Inhaler works reliably	With this attribute, we would like you to think about how important it is to have an inhaler that works well every time you use it. Inhalers that work well: ▪ Don’t clog ▪ Lock when all the medicine has been used ▪ Don’t accidentally release the dose when stored in your pocket or purse
Inhaler makes inhaling your medicine easy	With this attribute, we would like you to think about how important it is to have an inhaler that lets you inhale your medicine easily.
Having clear instructions to use your inhaler	With this attribute, we would like you to think about how important it is to have an inhaler that includes clear instructions and that are easy to follow.
Inhaler is durable	With this attribute, we would like you to think about how important it is to have an inhaler that continues to work after falling on the ground or going through an incident that could damage it.
Inhaler is easy to clean	With this attribute, we would like you to think about how important it is to have an inhaler that is easy to clean after you use it.
Inhaler is easy to use	With this attribute, we would like you to think about how important it is to have an inhaler that is easy to use as you take your medicine. With inhalers that are easy to use, you: ▪ Like the number of steps needed to take the medicine ▪ Like the time needed to take the medicine.
Medicine comes out of the inhaler at a comfortable speed for inhalation	With this attribute, we would like you to think about how important it is to have an inhaler that allows the medicine to come out at a comfortable speed so you can inhale your medicine easily.
Inhaler is easy to hold	With this attribute, we would like you to think about how important it is to have an inhaler that can be held without difficulty when taking your medicine.
Inhaler is easy to carry with you	With this attribute, we would like you to think about how important it is to have an inhaler that you can carry with you without difficulty.

The experimental design consisted of 20 BWS questions. Questions were divided into two 10-question blocks to reduce the number of BWS questions answered by each participant, which reduces measurement error and ensures that all questions in the design are presented across participants. Each participant was randomly assigned to answer 10 questions. The use of this design maximized the likelihood of having an identifiable statistical model to analyze responses to a set of 20 BWS questions [23].

#### Pretest of attribute definitions and BWS questions

Cognitive debriefing interviews were conducted before implementation of the online survey to evaluate the ease of completion and understanding of the instructions, inhaler attributes, and attribute definitions for the draft BWS questions. Cognitive debriefing interviewing is a well-established qualitative research methodology used to identify problems with questionnaire formatting, instructions, items and response options [24, 25]. Specifically, two experienced interviewers asked participants to complete the questionnaire while thinking aloud or describing their thought processes as they answered the draft questionnaire items. During each interview, one interviewer served as the primary interviewer, while the other took notes and monitored the need for additional questions or probes. The interviewers used a semistructured interview guide that included probe questions designed to help interviewers understand how each participant interpreted and chose their answers for each item in the draft questionnaire. The attributes were pretested through one-on-one, face-to-face cognitive debriefing interviews with five adult participants who had filled at least one prescription for Combivent Respimat for their respiratory condition.

All five participants were able to complete both blocks of 10 draft BWS questions with ease. Participants understood the majority of attributes and their definitions as written. Participants responded well to the approach of selecting the most important attribute and the least important attribute; thus, no changes were made to these anchors. PASAPQ item 7, size of the inhaler, was not included in the final attribute list because it overlaps with two other items (i.e., “inhaler is easy to carry with you” and “inhaler is easy to hold”). Based on participant feedback, minor changes were made to improve the clarity of the instructions and the formatting of the BWS questions.

### Data collection

Participants were identified and recruited for participation in the survey via VisibilityRx’s pharmacy network, following a process compliant with HIPAA [19]. The VisibilityRx network represents 26,000 retail, mail order, and specialty pharmacies, which fill more than 35% of the nation’s retail, mail order, and specialty pharmacy prescriptions [18]. The VisibilityRx deidentified pharmacy prescription database is diverse in terms of patient demographics, geographical location, and payer sources, as well as the type of pharmacy.

To be eligible for the study, participants must have met the following inclusion criteria based on the information gathered from the database and screener:

- Aged 40 to 75 years
- Filled at least two prescriptions for a Combivent Respimat inhaler from March 1, 2013, to August 31, 2013
- Able to understand and provide consent
- Able to complete in English

There were 19,964 people within the pharmacy network who had filled at least two prescriptions for Combivent Respimat within the specified time. No patient-level data regarding the age of the patient was available from the pharmacy network. Using a random selection process, VisibilityRx provided a de-identified sample list to the pharmacy network for reidentification of patients and generation of a patient letter. The pharmacy network then mailed letters to 8150 potentially eligible participants in waves timed 3 weeks apart (i.e., 4000 invitations sent in waves 1 and 2 and 150 invitations in wave 3). Each wave was sent to unique participants. The survey was closed once the target sample size of 500 was reached.

The invitations introduced the survey and provided a link to a secure website to access the questionnaire. Following electronic consent, eligibility was confirmed via screening questions and, if eligible, the participants were given access to the questionnaire. The sample excluded residents of California and Massachusetts due to local restrictions at the pharmacy level.

The web survey system was programmed using HTML5. This technology allows optimal user interface and user experience based on the size and rotation of the device being used by the participant.

### Analysis methods

Summary statistics were calculated for demographic and clinical background information. Available demographic information at the aggregate level from the pharmacy network database (i.e., age, sex, and drug payment type) was used to compare characteristics of participants who completed the survey (participants) and participants who did not participate or complete the survey (nonparticipants). The frequency and percentage of participants who completed each of the 14 PASAPQ item response options (i.e., 1 [very dissatisfied] to 7 [very satisfied]) and the extent of missing for each item were evaluated. Missing items were imputed using the PASAPQ domain mean from available responses when at least half of the items were nonmissing.

Unweighted descriptive statistics (means, standard deviations [SD], median, and range) of the 14 PASAPQ item scores, the total score, the performance domain score, and the convenience domain score were computed overall and by key patient characteristics (i.e., age, sex, number of Combivent Respimat inhaler prescriptions filled, frequency of use, employment status).

### Analysis of best-worst scaling questions

The identification of the most important and least important attribute in each question were treated as sequential decisions. To identify a model that considers both decisions, the utility (indicating relative preference) associated with the inhaler attribute selected as *most* important and the utility associated with the inhaler attribute selected as *least* important were constrained to each reflect the same change in utility, albeit in opposite directions. Because BWS questions provided two pieces of information (the most and the least important attribute in a set of inhaler attributes), each question resulted in two observations for each participant. For one observation, the most important inhaler attribute was given a value of 1. For the second observation, the least important inhaler attribute was given a value of −1. For both observations resulting from each question, the attribute that was not chosen as either most or least important was given a value of 0.

A main-effects random-parameters logit (RPL) regression model that relates participants’ choices for the most and least important attribute to all inhaler attributes in the full set of attributes was used to estimate a relative preference weight for each attribute [26]. For identification purposes, the mean importance of the inhaler attributes in the question was normalized to have a utility of 0. RPL controls for the correlation of multiple responses from the same participant (i.e., the most important attribute and least important attribute) for each BWS question, as well as the correlation of responses from the same participant over the series of questions [26, 27]. Finally, to produce the final set of relative importance weights, preference-weight estimates from the model were transformed into relative attribute importance weights using probability-based rescaling [28, 29]. The relative importance weight indicates the importance of any inhaler attribute relative to the importance of the attribute identified as the most important among all attributes.

To investigate whether relative importance varies by observable participant characteristics (e.g., number of Respimat inhalers used), we estimated models for select mutually exclusive subgroup pairs and compared the relative importance weights between the subgroups in the pair. Four subgroup pairs were considered:

- Participants age 40 to 59 versus age 60 to 75 years
- Participants being male versus female
- Participants using two or three Combivent Respimat inhalers versus using four or more inhalers in the last 8 months
- Participants using their inhaler with a specific frequency every day based on a fixed dosing (once or twice or “other”) versus participants who reported using their inhaler as needed

To conduct this comparison, we first created a dummy variable (i.e., 1 when the participant belonged to one subgroup in a pair; 0 when the participant belonged to the other subgroup in a pair) and interacted the dummy-coded variable with all explanatory variables (i.e., attributes) in the RPL model. A test of the joint significance of the interaction terms (a Wald chi-square test) was used to determine whether there were systematic differences in the relative importance weights between the two subgroups in each pair. Any participant who answered at least one BWS question was included in the analysis.

## Results

### Study sample

**Fig. 2:**
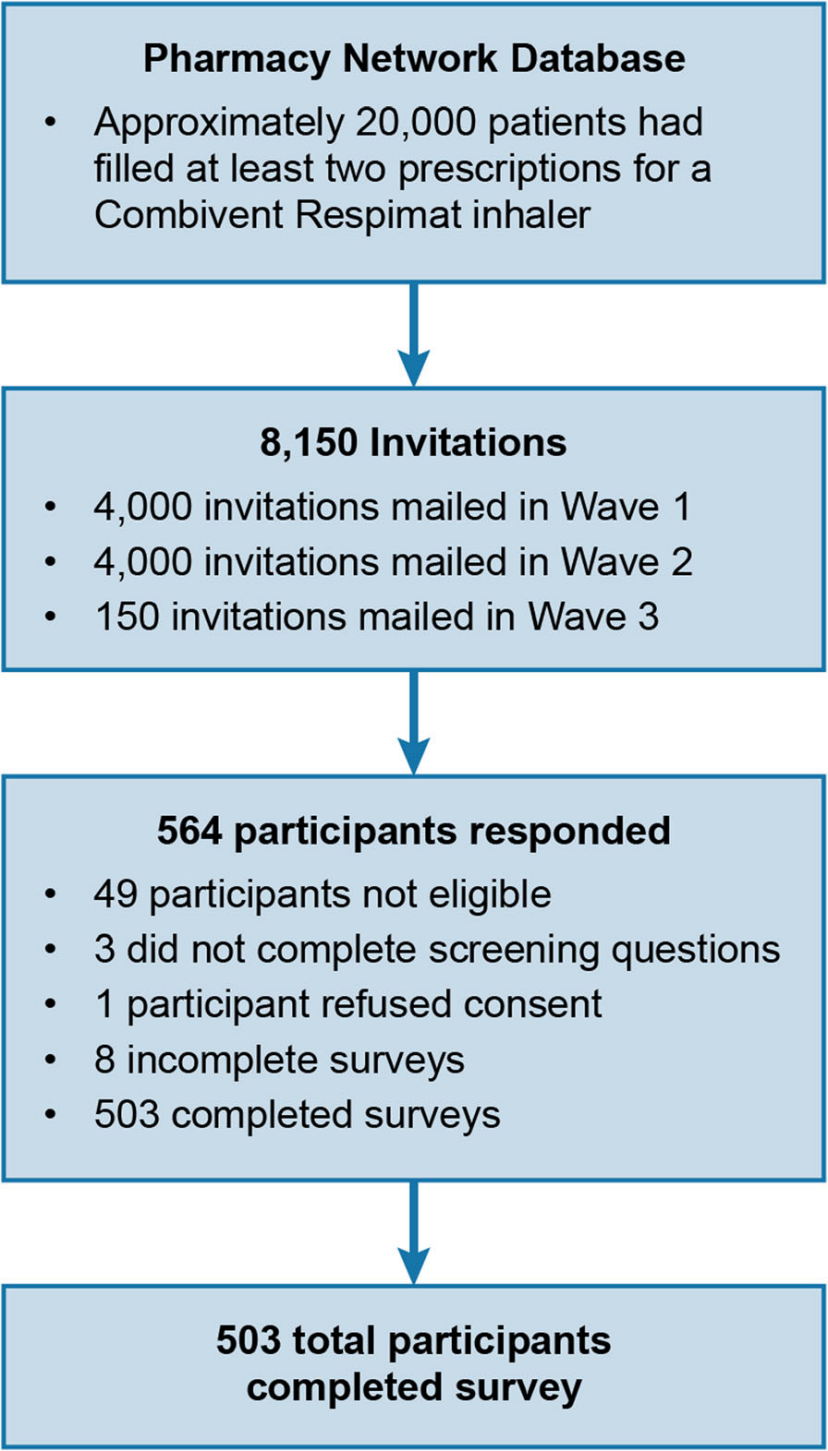
Study Flow Chart

Invitations were mailed to 8150 potentially eligible people. There were 564 participants who started the questionnaire. Of these, 3 did not complete the screening questions, 49 were determined ineligible, 8 did not complete the questionnaire, and 1 refused consent. Therefore, 503 of the 564 participants who started the screener (89.2%) completed the questionnaire. Recruitment ended once the targeted sample size of 500 was reached (Fig. 2).

Table 3 summarizes participants’ demographic and clinical background information. The majority of participants were female (57.3%), white (88.5%), and 51 to 70 years old (67.6%). Approximately 36% completed high school and 49.1% attended college. Approximately 71% reported being currently unemployed or retired. Among participants, 46.9% had a diagnosis of COPD only, 21.9% had asthma only, 8.2% had another lung disease, and 23.1% had more than one lung disease.

**Table 3:** Demographics and clinical information (*N* = 503)

Characteristic	n (%)
Sex	
Male	215 (42.7)
Female	288 (57.3)
Age	
40 to 50 years	108 (21.5)
51 to 60 years	181 (36.0)
61 to 70 years	159 (31.6)
71 to 75 years	55 (10.9)
Race	
White	445 (88.5)
Black/African American	33 (6.6)
American Indian or Alaska Native	7 (1.4)
Native Hawaiian	1 (0.2)
Other Pacific Islander	0 (0.0)
Asian	4 (0.8)
Other	12 (2.4)
Highest grade completed	
Less than High School	74 (14.7)
High School Diploma or equivalent (e.g., GED)	182 (36.2)
Some College	119 (23.7)
College Degree	82 (16.3)
Professional or advanced degree	46 (9.1)
Employment status	
Employed full-time	114 (22.7)
Employed part-time	28 (5.6)
Not employed or retired	358 (71.2)
Number of Combivent Respimat prescriptions over past 8 months	
2 inhalers	143 (28.4)
3 inhalers	100 (19.9)
4 or more inhalers	260 (51.7)
Taking Combivent Respimat for:	
Chronic obstructive pulmonary disease only	236 (46.9)
Asthma only	110 (21.9)
Other lung disease only	41 (8.2)
More than one disease	116 (23.1)
Using the Combivent Respimat for...	
Less than 3 months	68 (13.5)
3 to 6 months	188 (37.4)
7 to 12 months	179 (35.6)
I don’t know	68 (13.5)
Take Combivent Respimat...	
Once daily	20 (4.0)
Twice daily	120 (23.9)
Other	95 (18.9)
As needed	268 (53.3)
Ever used other inhalers?	
Yes	426 (84.7)
No	77 (15.3)
Ever used nebulizer	
Yes	310 (61.6)
No	157 (31.2)
I don’t know	36 (7.2)

Nearly three quarters of participants (73%) reported using the Combivent Respimat inhaler for 3 to 12 months, and 51.7% reported using four or more Combivent Respimat inhalers over the previous 8 months. Approximately 53% indicated they were using their inhaler on an as-needed basis.

The 503 participants and the 7647 nonparticipants had similar distributions across age, sex, and type of insurance (i.e., no insurance, private third party, Medicaid or Medicare).

### Satisfaction scores

Most participants reported being satisfied or very satisfied with the Respimat device as measured by PASAPQ items, ranging from 61% on PASAPQ item 2 (feeling that the inhaled dose goes to lungs) to 75% on PASAPQ item 8 (inhaler is durable) (data not shown for the individual PASAPQ items). Up to 20% reported being somewhat dissatisfied to very dissatisfied on any PASAPQ item. Only 7.4% reported being very dissatisfied on item 10 (with using the inhaler) and item 14 (overall satisfaction with inhaler). Missed responses were exceptionally low; no single item had more than one missing response.

The mean (SD) of the total, performance, and convenience scores were 75.0 [22.3], 74.4 [24.2], and 75.8 [21.9], respectively, indicating that participants were highly satisfied with Combivent Respimat (Table 4). These results show that satisfaction levels were not statistically different across the domains in the PASAPQ (*P* > 0.05). Additionally, the median total, performance, and convenience scores were approximately 7 points higher (82.1, 81.0, and 83.0, respectively) than their mean scores (data not shown). In general, the average (and median) scores were comparable across the levels of key patient characteristics (Table 4). Notably, higher average and median total, performance, and convenience scores, indicating relative greater satisfaction, were observed for younger participants (aged <70 years) versus older participants (≥ 70 years), and for participants who were unemployed, retired, or employed part-time versus participants employed full-time.

**Table 4:** Descriptive statistics of PASAPQ total score, performance, and convenience domains by key information^a^

	PASAPQ Total Score^b^ Mean (SD)	PASAPQ Performance Domain Mean (SD)	PASAPQ Convenience Domain Mean (SD)
Overall (*n* = 503)	75.0 (22.3)	74.4 (24.2)	75.8 (21.9)
Age			
40 to 50 years (*n* = 108)	76.4 (23.1)	76.8 (23.0)	76.0 (24.4)
51 to 60 years (*n* = 181)	77.0 (21.5)	76.3 (23.7)	77.7 (20.3)
61 to 70 years (*n* = 159)	74.1 (21.3)	72.8 (23.8)	75.5 (20.9)
71 to 75 years (*n* = 55)	68.6 (25.0)	67.6 (27.8)	69.6 (24.2)
Sex			
Male (*n* = 215)	75.6 (20.5)	75.9 (21.4)	75.1 (20.9)
Female (*n* = 288)	74.6 (23.6)	73.2 (26.0)	76.3 (22.7)
Number of Combivent Respimat prescriptions over past 8 months			
2 inhalers (*n* = 143)	74.8 (22.9)	74.4 (24.7)	75.2 (22.8)
3 inhalers (*n* = 100)	75.0 (19.9)	74.9 (21.5)	75.1 (19.6)
4 or more inhalers (*n* = 260)	75.2 (22.8)	74.2 (25.0)	76.3 (22.3)
Take Combivent Respimat...			
Once daily (*n* = 20)	78.7 (19.2)	80.0 (20.0)	77.2 (19.9)
Twice daily (*n* = 120)	77.4 (22.4)	77.1 (23.3)	77.7 (22.8)
Other (*n* = 268)	73.4 (22.5)	72.5 (24.5)	74.5 (22.2)
As needed (*n* = 95)	75.7 (22.0)	74.9 (24.9)	76.7 (20.3)
I don’t know (*n* = 68)	71.4 (21.9),	70.1 (23.5)	72.8 (21.5)
Ever used other inhalers			
Yes (*n* = 426)	74.5 (22.7)	73.7 (24.7)	75.5 (22.2)
No (*n* = 77)	77.7 (19.9)	77.9 (20.7)	77.5 (20.3)
Employment status			
Employed full-time (*n* = 114)	68.2 (25.7)	68.2 (27.5)	68.2 (25.3)
Employed part-time (n = 28)	76.4 (23.9)	76.8 (24.6)	75.9 (24.6)
Not employed or retired (*n* = 358)	76.9 (20.6)	76.0 (22.7)	78.0 (20.1)

*PASAPQ* Patient Satisfaction and Preference Questionnaire, *SD* standard deviation

^a^Scores calculated using imputed values. Missing items were imputed using the domain mean from available responses when at least half of the items were nonmissing

^b^The total score for the PASAPQ is derived from a summary of the first 13 questions

### Relative importance of inhaler attributes

**Fig. 3:**
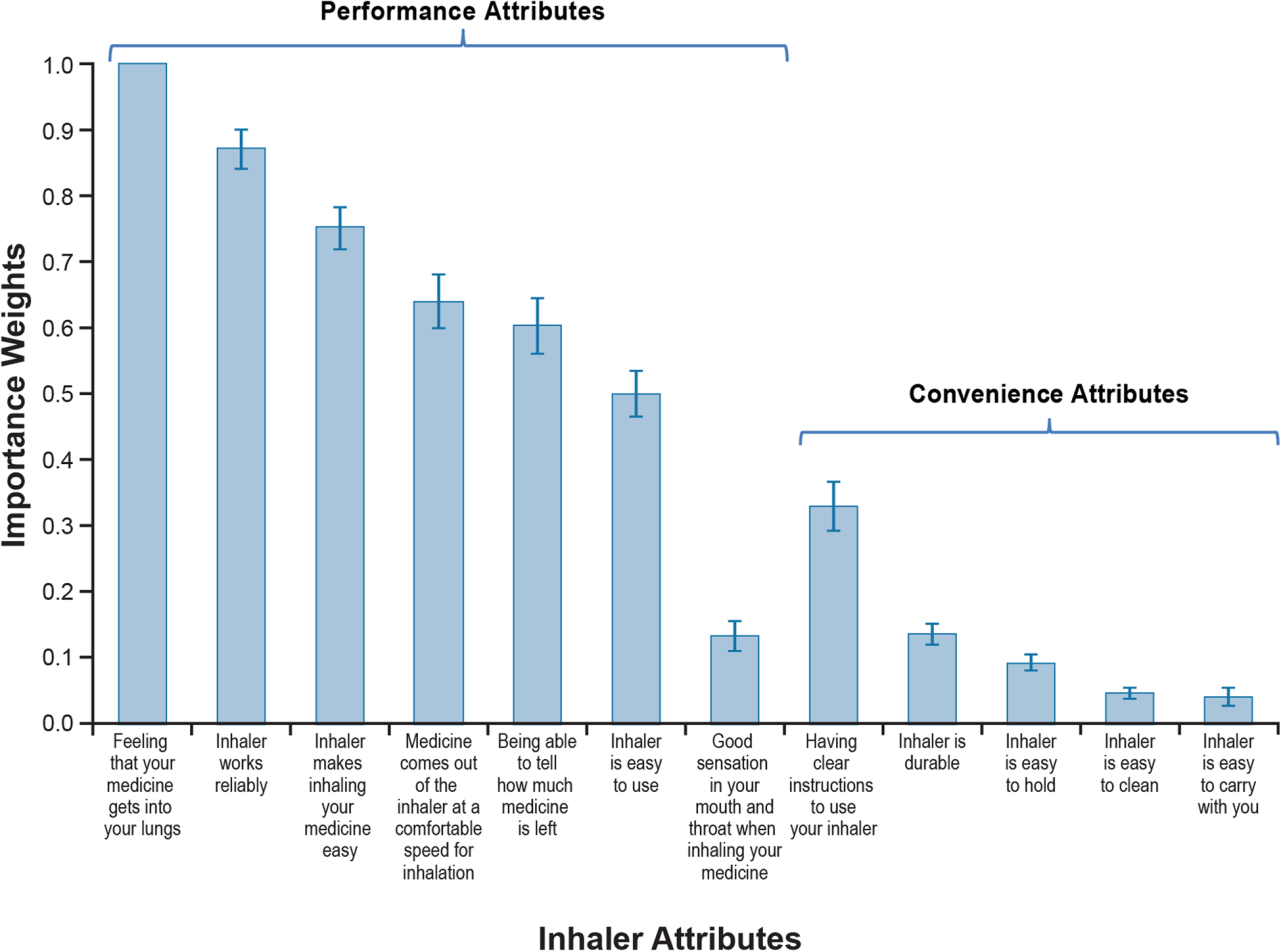
Importance Weights for Performance and Convenience Attributes for the Full Sample (*N* = 503). Note: The vertical bars surrounding each mean importance weight denote the 95% CI about the point estimate

Figure 3 summarizes the relative importance of the inhaler attributes included in the study. Inhaler attributes are presented in order of decreasing importance with each of the corresponding domains in the PASAPQ (i.e., performance and convenience). Overall, *Feeling that your medicine gets into your lungs*, *Inhaler works reliably, Inhaler makes inhaling your medicine easy*, *Medicine comes out of the inhaler at a comfortable speed for inhalation*, *Being able to tell how much of your medicine is left*, and *Inhaler is easy to use* were the six most important attributes and correspond to items in the performance domain in the PASAPQ. Among the attributes that correspond to the convenience domain of the PASAPQ, *Having clear instructions to use your inhaler* was the most important. The least important inhaler attribute was associated with an item in the convenience domain, *Inhaler is easy to carry with you*.

The vertical lines on each of the relative importance weight bars show the 95% confidence interval (CI), and nonoverlapping CIs indicate statistically significant differences (*P* < 0.05). For example, *Inhaler is durable* was statistically significantly less important than *Having clear instructions to use your inhaler* but was not statistically significantly different from *Good sensation in your mouth and throat when inhaling your medicine*.

**Fig. 4:**
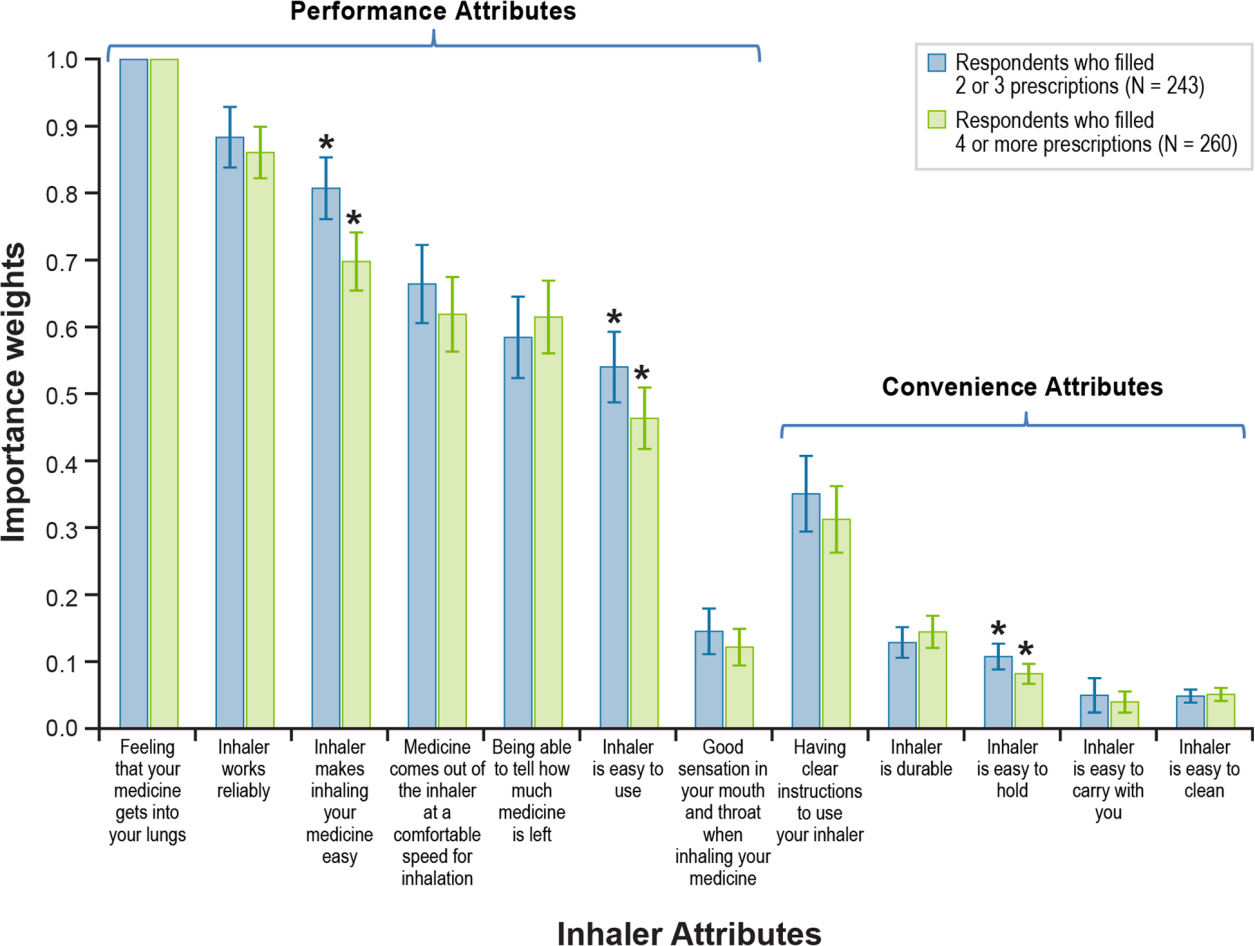
Importance Weights for Performance and Convenience Attributes Stratified by Previous Inhaler Use. Note: The vertical bars surrounding each mean importance weight denote the 95% CI about the point estimate. *Statistically significantly different from the importance weight in the other subgroup category

Among the four subgroup pairs evaluated in this study, systematic differences in preferences were detected only between participants who reported using two to three inhalers during the past 8 months and participants who reported using four or more inhalers (*P* < 0.01). Figure 4 presents the estimated relative importance weights for these two subgroups. Two attributes related to Performance (*Inhaler makes inhaling your medicine easy* and *Inhaler is easy to use*) and one attribute related to convenience (*Inhaler is easy to hold*) were less important to participants who had used four or more inhalers than to those who had used fewer inhalers. Relative importance weights were not statistically different between the subgroup pairs based on age, sex, or frequency of inhaler use.

## Discussion

This study provides valuable information about the relative importance of inhaler attributes corresponding to items in the PASAPQ. Variation in the mean estimates of the importance weights and the narrow CIs around the mean estimates suggests that study participants had well-defined preferences for the inhaler attributes included in the survey.

The results from this study also highlight the differences between satisfaction and preferences. Although we found no statistical differences in participants’ satisfaction reported under the performance and convenience domains, there were significant differences in relative preferences for attributes corresponding to items between the two domains. Perhaps not surprisingly, the study participants stated that attributes corresponding to items in the performance domain were generally and significantly more important than those corresponding to items in the convenience domain.

Measuring satisfaction reliably provides important information for the comparison of inhalers based on patients’ experiences with inhalers. However, our results suggest that changes in patients’ well-being associated with differences in satisfaction across inhalers may depend on the items for which users report differences. Moreover, given the significant differences in preferences for attributes corresponding to items in the PASAPQ, if a treatment could improve satisfaction on the attributes of an inhaler that are deemed most important to a patient, then the treatment may potentially have a greater impact on the patient’s well-being.

Up to 20% of participants reported being somewhat dissatisfied to very dissatisfied on any PASAPQ item. Data were not collected to determine the reasons for satisfaction or dissatisfaction. Participants who reported dissatisfaction may have had long-term experiences with the use of a metered-dose inhaler device and may have needed more time to adjust and get familiar with the use of a new device. Participants may have experienced a recent worsening of their lung condition and were dissatisfied due to worsening symptoms. In addition, satisfaction with any device may also depend on patient-level characteristics (e.g., age, severity level of lung condition, cognitive functioning, other comorbid conditions, employment), which were not collected in the study.

Approximately 60% to 72% of participants reported being satisfied or very satisfied with the Combivent Respimat inhaler based on the six most important attributes related to the performance domain: *Feeling that your medicine gets into your lungs* (item 2), *Inhaler works reliably* (item 4), *Inhaler makes inhaling your medicine easy* (item 5), *Medicine comes out of the inhaler at a comfortable speed for inhalation* (item 11), *Being able to tell how much of your medicine is left* (item 3)*,* and *Inhaler is easy to use* (item 10). Importance weights for the most important attributes were largely statistically significantly different from each other, while importance weights for the least important attributes were not. Overall, there appears to be a clear demarcation of importance related to attributes associated with perceived treatment effectiveness compared with the other attributes.

The patterns of relative importance estimated for the overall sample were consistent across most subgroups evaluated in a post hoc analysis. Only importance weights among participants who reported filling two or three prescriptions versus those who reported filling four or more prescriptions were found to be statistically significantly systematically different. We speculate that differences in importance weights correspond to differences in patients’ familiarity with the inhaler. The implication of such a result is that treatment experience influences patients’ preferences. Despite differences in the estimated importance weights between these two subgroups, the same six attributes were most important to both subgroups.

The primary strength of this study is that the design reflects a real-world context where a participant may or may not have received any type of inhaler instruction; whereas the majority of prior studies that examined satisfaction with inhaler devices were clinical trials in which participants were typically given instructions [1, 2, 4, 8, 17]. Another important strength of the study is the ability to draw a diverse sample of participants from the VisibilityRx database rather than rely on self-reporting of prescription use.

There are limitations of the study design. The sample was a convenience sample intended to represent patients who are taking Combivent Respimat. The sample was diverse based on age, gender, and education, though less so based on race as the majority of participants were white. Most of the participants were unemployed or retired, which is consistent with the overall COPD population [30]. Participants were self-selected into the study, and participants with comorbid conditions were not accounted for, which may introduce biases in the measurement of satisfaction and importance of device attributes. The survey did not include any questions to measure the severity of respiratory impairment. The results may have been influenced by the treatment effect of Combivent, the participants’ experiences and satisfaction with the Respimat device as well as other experiences related to their disease, other comorbid conditions, cognitive functioning or other treatments that were not measured in the study. The results are not generalizable to new users of the Combivent Respimat or users of other devices.

A web-based survey may be considered a potential limitation. While certain aspects of Internet adoption by minorities, adults with lower socioeconomic status, and seniors are historically low and have not changed significantly since 2000, [31, 32] the Internet population has increased to 85% of adults in 2012 [31–33]. Internet surveys are generally accepted as an appropriate method of participant identification and survey administration [34].

While BWS questionnaires increasingly are used to evaluate the relative importance of outcomes or features of clinical interventions, the method has some limitations. Importance is not inferred from clinical choices made in the real world but rather from responses to designed scenarios under experimental control. Stated choices in BWS questions do not have the same consequences as treatment decisions. For this reason, differences can arise between the stated ranking in the BWS exercise and actual choices between devices that meet some or all of the attributes in this study.

The results of this study were compared with the results from the 48-week open-label, longitudinal study (*N* = 470) for patients in the Combivent Respimat arm [17]. The change between baseline and 48 weeks in the PASAPQ performance domain was used as a primary outcome. The overall mean performance score in this observational study (74.4) differs between 10 to 13 points from the adjusted mean performance score in the open-label study’s Combivent Respimat arm (range, 84.6 at 3 weeks to 87.9 at 48 weeks). This difference fell near the 10-point threshold characterized as a conservative minimally important difference [10, 11]. However, the observational study’s overall median performance score (81.0) was considerably closer to the adjusted average means observed in the open-label study. Comparisons were challenging for multiple reasons. The open-label study reported results at multiple time points, and only mean scores were reported. In the current study, total and domain score distributions were positively skewed toward higher PASAPQ scores.

Previous studies have elicited preferences for items included in a patient-reported outcomes instrument [35–39]. Patient-reported outcome questionnaires such as the PASAPQ are often scored by summing the values of each item to achieve a total score, which implies that each item in the PASAPQ has equal importance. The results of this study suggest that this assumption may not be true. Thus, understanding the relative importance of inhaler attributes may provide more insight into the impact of treatments on patient well-being.

This research has identified inhaler qualities that clinicians could use in discussions with a patient for decision making regarding treatment options. Among treatments with similar effectiveness, these qualities may make a difference to patients in terms of treatment adherence and clinical outcomes (e.g., “We have several options. This one is more reliable but less convenient, is that important to you?”). In addition, patient-reported satisfaction with an inhaler device is important, especially in relation to the performance attributes. Satisfaction with an inhaler device may contribute to the effectiveness of the treatment and therefore increase patients’ treatment outcomes, which may improve adherence and result in further benefit of therapy. This research provides evidence to support a patient-centric approach to treatment decision making.

Future research should explore patient satisfaction and preferences in terms of attribute importance in relation to adherence and improved clinical outcomes (e.g., frequency of exacerbations, hospitalizations), which might be an indicator of compliance with their treatment. Results from a systematic literature review of studies that evaluated the proper use of dry powder inhalers in patients with COPD or asthma noted that 4% to 94% of patients do not use their dry powder inhalers correctly [40]. Incorrect procedures included challenges with the devices such as incorrect dose metering, inhaler positioning, sequencing of the inhaler, and improper mouthpiece positioning [40]. Thus it is important to understand which attributes are important for patients. In addition, we recommend that future research evaluate preferences and satisfaction with inhaler attributes across patients who use different inhaler devices.

## Conclusion

The majority of participants reported being satisfied or very satisfied with the Combivent Respimat device across the PASAPQ items. Performance attributes were of greater importance than convenience attributes based on this patient survey for the Combivent Respimat device.

## Abbreviations

BWS: best-worst scaling
CI: confidence interval
COPD: chronic obstructive pulmonary disease
PASAPQ: Patient Satisfaction and Preference Questionnaire
RPL: random-parameters logit
RTI-HS: RTI Health Solutions
SD: standard deviation
